# Paroxysmal hemicrania-tic syndrome: a new case report

**DOI:** 10.1186/1129-2377-14-S1-P50

**Published:** 2013-02-21

**Authors:** I Maestrini, A Viganò, G Di Stefano, A Truini, G Cruccu, GL Lenzi, V Di Piero

**Affiliations:** 1 Department of Neurology and Psychiatry, ‘Sapienza’ University of Rome, Rome, Italy

## Introduction

An association of Paroxysmal Hemicrania (PH) with Trigeminal Neuralgia (TN) was described in eight patients [1,2] and has been called the PH-tic syndrome [3]. Case report. A 52-year-old man presented with a 5-year history of excruciating and burning pain, involving the left ophthalmic trigeminal branch, lasted 30 to 60 seconds, occurred 2 to 5 times a day, without any autonomic sign. Triggering factors included touching, washing face or brushing the teeth. Carbamazepine (600 mg/day) produced marked improvement. Any attempt to reduce the dose resulted in pain recurrence. While the previous pain was in remission by carbamazepine, he complained of a second type of pain that lasted 15 to 30 minutes and occurred up to 8 times per day. The strictly unilateral pain occurred in the left orbit, forehead, temple, nose and was described as severe and sharp with autonomic signs. There were no triggers. Indomethacin (150 mg/day) completely resolved attacks. His past medical history was significant for atrial fibrillation, hypertension and glaucoma; neurological examination and blood analysis were normal. Brain magnetic resonance showed silent lacunar infarcts, while magnetic cerebral angiography was normal. Trigeminal reflexes were also normal. In order to assess the possible involvement of the small myelinated and unmyelinated trigeminal fibers, we recorded, using a Nd:YAP laser stimulator, laser evoked potentials (LEPs) after supraorbital stimulation that showed a normality of A-delta and C fibers activation of the affected compared to the normal side. At the time of LEPs the patient was TN and PH off-indomethacin pain-free.

## Conclusion

LEPs study supported the diagnosis of TN idiopathic. It is still debated if the rare association of TN and PH is a new entity or two distinct disease. Further investigations by neuroimaging might be useful to clarify this issue and to better understand the pathophysiology of these entities [4].
